# Vital yet Fragile: Informal Networks of Support Among Young People Navigating Long Covid

**DOI:** 10.1111/hex.70611

**Published:** 2026-03-11

**Authors:** Zaira Clarke, Hannah Cowan, Tim Rhodes, Praveena Fernes, A. Haines, Lucinda Leal

**Affiliations:** ^1^ London School of Hygiene and Tropical Medicine London UK; ^2^ Peer Researcher UK

**Keywords:** alternative care, care networks, Covid‐19, invalidation, Long Covid, medical gaslighting, validation, young people

## Abstract

**Introduction:**

Young people living with Long Covid face challenges accessing health care and social support. Previous qualitative research in the UK has described the ‘invalidation’ of Long Covid illness experience. It has been said that there is a ‘double invisibility’ produced by narratives that minimise the effects of Covid‐19 among young people, which combine with a generalised lack of awareness of Long Covid itself. In this analysis, we look beyond the well‐documented networks of online self‐help and advocacy to trace how young people navigate, connect and maintain multi‐sited alternative care networks to manage their everyday experiences of Long Covid.

**Methods:**

We draw on the analysis of qualitative interviews with 54 young people aged 15–25 with long‐term health impacts from Covid‐19, of whom 30 also participated in follow‐up interviews. The sample includes young people with multiple genders, who identify with a range of ethnic identities, and who have experience of neurodiversity or additional disabilities. Interview transcripts were analysed to identify key themes, in collaboration with a group of peer researchers who are co‐authors on this study.

**Results:**

We find that the informal networks that are navigated and created by young people play a vital role, but that they are also fragile. We present our findings across four themes—how informal networks afford young people validation in different ways; the material differences informal networks bring to young people's lives; the work that young people do to build and maintain these networks; and the fragility of support networks. We show that informal networks are not simply identified and found, but that they are ‘made to work’ by young people who do the work that brings informal networks together and that holds them in place.

**Conclusion:**

We conclude that there is a need to strengthen the vital work of informal care that is done by young people, but that alternative care networks should not be seen simply as a means of ‘filling the gaps’ of inadequate care. There is a need to build infrastructures that properly integrate formal with informal care in direct response to young people's experiences of Long Covid.

**Patient or Public Contribution:**

This qualitative study was undertaken in close collaboration with community partners and co‐produced with young people affected by Long Covid, using participatory methods. Young people affected by Long Covid were involved in a series of consultations, workshops and meetings focused on the analysis of data and their development into project outputs, including as authors of this paper.

## 1 Introduction

Long Covid is characterised by fluctuating and debilitating symptoms that are multiple and cross‐system, including chronic fatigue, brain fog, and cardiovascular, gastrointestinal and neurological symptoms [1, 2]

The term Long Covid emerged during the pandemic, as individuals connected online to share their experiences of Covid‐19 infections persisted beyond short‐term mild illness. Outpacing formal medical understandings of Covid‐19, patient testimonies were promoted through hashtags and digital advocacy efforts and subsequently discussed in academic publications and parliamentary debates, transforming Long Covid into a ‘recognisable “scientific object”’ [3]. Yet many people affected have struggled to access care and support in the face of scepticism about the veracity of Long Covid illness experience [4, 5], with patients discredited as ‘unreliable informants of their own illness experiences’ [6].

Young people living with Long Covid have had particular difficulty in accessing formal healthcare services [4]. In the UK, the lack of access to care is not only limited by National Health Service (NHS) staff shortages and insufficient funding [7], but by widespread narratives early in the pandemic that children and young people were not at significant risk from Covid‐19 [4]. Whilst young people became a social and cultural focus in pandemic narratives, labelled the ‘Covid Generation’ [8], this was largely focused on the social, psychological, and economic effects of altered transitions into adulthood. Young people with Long Covid have experienced pronounced alterations to expected transitions to young adulthood, such as finishing school, living independently, starting work, or parenthood [9, 10]. With formal care often inaccessible, fragmented or insufficient, young people must find alternative routes to get by in response to chronic ill health. This study looks beyond the well‐documented networks of online advocacy [6, 11] to trace how young people navigate, connect, and maintain multi‐sited alternative care networks to manage everyday experiences of illness.

### 1.1 Invalidation in Formal Care Settings

Ideas of ‘illness invalidation’ are not new, and have long been documented in relation to chronic and unfolding conditions [12, 13, 14, 15]. A key theme in previous qualitative research on Long Covid has been the ‘invalidation’ of illness experience in the face of medical uncertainty, conflicting expertise, and fragmented care, resulting in patients being ‘dismissed’ [11], ‘gaslighted’ [16], and ‘fobbed off’ [17] by medical professionals. The multiple reactions from medical professionals illustrate that illness (in)validation is always a matter of social interaction and experience, and never simply constituted by a singular consensus or a gold standard of expertise. Maclean et al. [5] build on the concept of patient candidacy used more broadly by Dixon‐Woods et al., [18] to trace how patients with Long Covid have their illness candidacy ‘rejected’ or ‘diverted’, resulting in them being ‘abandoned’. In contrast, when illness candidacy is ‘validated and affirmed’, healthcare interactions were ‘more positive’, even in the face of ‘extreme uncertainty about the causes, consequences and prognosis for long Covid’ [5]. Wild et al. [4] illustrate how this candidacy is shaped by the lack of ‘social currency’ for young people with Long Covid in particular, formed through dominant popular discourses which both minimise the severity of Long Covid, as well as the impact of Covid‐19 on young people, resulting in a ‘double invisibility’. These narratives can accentuate doubt through characterising parents as overprotective and young people as unreliable narrators, undermining access to recognition, care and support.

How illness experience is recognised and understood is a critical factor shaping care, with illness invalidation ‘ultimately hindering healthcare service utilisation’ [19]. Firstly, illness invalidation can reproduce relations of distrust in services, as well as introduce internalised self‐doubt and disorientation when attempting to navigate difficult‐to‐access care [4, 6, 11, 19, 20, 21]. Secondly, uncertainty in relation to Long Covid diagnosis, experience and expertise has also shaped service responses [2, 5]. While, for instance, the NHS signalled a commitment to deliver combined physical, clinical and psychosocial care through establishing Long Covid clinics [7], more recently some of these initiatives have been merged with other services, scaled back or closed, with young people said to have been ‘abandoned’ [22]. Whilst many have found experiences of validation in Long Covid clinics, lengthy waiting times leave many young people to manage their symptoms alone and unable to provide evidence to access reasonable adjustments at school [23]. Given the unevenness of formal care that people with Long Covid experience [17, 20], alternative and informal networks of support become vital [24].

### 1.2 Informal Care Networks

The Long Covid literature to date suggests that online, informal and peer support act as alternative mechanisms of ‘reassurance’, ‘recognition’ and ‘collective gathering’ in response to ‘a shared account of rejection by the healthcare system’ [3, 11, 17, 25]. These informal care networks are largely celebrated for their capacity not only to bridge ‘gaps’ in care provision and expertise [11, 26], but as having ‘sociopolitical’ potential in bringing about ‘collective gatherings’ which are ‘transformatory’ [27]. Yet it is important to note that alternative and informal networks of care in relation to Long Covid are themselves often fluid and precarious. Some scholars illustrate a lack of engagement among those most socially and economically marginalised, potentially because of the lack of relatability within peer groups, which majority middle‐class white women [28], whilst others draw attention to competing or uncertain information which circulates in these networks [11, 17]. This literature, however, continues to centre on online advocacy networks, as well as on adults with Long Covid when understanding alternative care.

In this qualitative analysis, we consider the multiple forms of connection that young people draw upon as well as build in their navigation of Long Covid care and support across multiple social contexts. We view care as a practice [29], which is made up of networks of multiple human and non‐human actors, which often entangle across more ‘formal’ or ‘informal’ settings [30]. We recognise that the boundaries between ‘formal’ and ‘informal’ care may not always be clearly defined, but for our purposes here we focus on forms of care that are not usually considered within the clinical encounter [30]. Our analysis maps how young people identify, harness and actively make alternative and informal networks of care which have emerged in response to the limits (and harms) of formal state‐organised biomedical care. Informal forms of care have been transformative in the face of a lack of formal care during the Covid‐19 pandemic, particularly for those who are marginalised and who may have more difficulty accessing formal services [31]. Whilst marginalised communities may have benefitted less from online Covid‐19 support networks, evidence suggests that other forms of alternative or peer support may be more inclusive [28]. We also draw on wider chronic health literature to describe how creating and sustaining alternative and informal networks of care takes *work*. The efforts to navigate and make care in and amongst informal networks can also bring about invalidation of illness [4], and can become exhausting in itself, exacerbating the effects of Long Covid and of chronic fatigue [32, 33, 34]. Moreover, informal care networks are both products of and effects of their structural conditions, which can limit as much as drive their potential. In this study, we therefore sought to explore informal or ‘alternative’ care networks that are active in the worlds of young people with Long Covid, especially those that become more important when formal state care fails. We look not only at who and what is involved in these networks, but also how they manifest, how they are engaged with, and what benefits or difficulties they bring into young people's lives.

## 2 Methods

We explore the alternative support networks of young people with Long Covid, drawing on analyses of qualitative interview data generated as part of a UK‐wide qualitative longitudinal study with young people aged 15–25 years. Understanding informal care networks was a key aim of our study. Using in‐depth semi‐structured interviews, we sought to understand the composition of these networks, how they are lived, established, and enacted, and what benefits and potential difficulties they bring to the lives of young people with Long Covid. We also engaged in co‐production through consultations and participatory methods with young people to explore how experiences of Long Covid intersect with health, well‐being, social life, inequalities and futures. Ethical approval was granted through the London School of Hygiene and Tropical Medicine Observational Research Ethics Committee, and via the Research Ethics Committee of Imperial College, linked to participant recruitment (see below).

### 2.1 Co‐Production

We engaged young people throughout the project after funding was secured. In the data generation stages, we engaged 33 young people with Long Covid and those impacted in broader ways by Covid‐19 and the pandemic through a series of eight consultation sessions run in collaboration with Long Covid Kids (a charity), Long Covid Support (a charity) and community youth organisations working with young people in London and nationwide. The young people who attended provided feedback which informed our recruitment methods, interview guide, and approach to engaging young people experiencing chronic illness and disability. In the data interpretation and analysis stages, we developed an advisory group of 10 paid peer researchers. We have undertaken 14 workshops and meetings with this advisory group so far, where they have reviewed excerpts of interview transcripts and supported the interpretation and analysis of interview data, including around informal networks. We then engaged with two peer researchers (L.L. and A.H.) who contributed as co‐authors through three stages: helping to generate themes in group discussion; reviewing and discussing an early draft; and reviewing a final draft and commenting before submission.

### 2.2 Interview Recruitment

Given fluctuating and unfolding understandings of Long Covid, we took a broad approach to recruitment, focusing on those aged 15–25 to look specifically at young people over their transition into adulthood. In addition to young people diagnosed with, or reporting, Long Covid and/or ongoing or persistent symptoms following Covid‐19, we also included some young people with experiences of ongoing health impacts from Covid‐19 and the pandemic more broadly to explore the way many young people's experience of Long Covid has been invalidated by medical professionals. This study focuses on 54 interview participants (of whom 30 participated in follow‐up interviews), which is a subsection of our overall sample of 72 participants who participated in the study. These 54 participants all:

a. have a Long Covid diagnosis (23 baseline; 18 follow‐up);
b. report Long Covid experience but are self‐diagnosed (15 baseline; 6 follow‐up); or
c. describe persistent and ongoing health effects related to SARS‐CoV‐2, but are unsure whether to describe this as Long Covid (16 baseline; 6 follow‐up).

For the purposes of this analysis, we do not include participants who described their health and well‐being as affected by the pandemic more broadly in other ways (18 baseline; 0 follow‐up). Our analysis here, therefore, focuses on those experiencing ongoing symptoms at baseline (54) and at follow‐up (30).

Given socioeconomic, ethnic, and gender disparities in Long Covid diagnosis and experience [28], we also sought to reach a diverse demographic of participants. We recruited these 54 participants in collaboration with a cohort study called REACT at (Imperial College London) (*N* = 39), Long Covid Kids (a charity) (*N* = 9), Long Covid Support (a charity) (*N* = 3) and community youth organisations (*N* = 3), specifically calling for those ‘who feel like they experience some form of inequality or uncertainty in their lives’. Potential participants in the REACT study were emailed directly from the Imperial College London team, contacting only those aged 15–25 who were reporting ongoing symptoms for at least 12 weeks after Covid‐19 infection. We ended up reaching out to all participants in this group, but began by reaching out to those in the lowest quartile of the Index of Multiple Deprivation, and with marginalised ethnic identities. Participants from all recruitment routes opted in by emailing the research team and were sent a project information sheet outlining the purpose of the research. Those who wanted to participate had at least 24 hours to read the information sheet and complete a consent form, as well as a form where they could self‐describe their positionality across a number of identity categories. We invited participants to a follow‐up interview based on who had been most impacted by their illness experience, whether the health impacts described during baseline interviews were ongoing, and whether they experienced a form of inequality.

Table 1 summarises the sample. Of the 54 participants included here, 30% (16) are aged under 18, 28% (15) are 18–21 and 42% (23) are 22–25, and we have heard from young people with multiple genders, who identify with a range of ethnic identities, and have experience of neurodiversity or additional disabilities. We note that our study includes a higher proportion of white female participants, 44% (24), despite efforts to maximise the diversity of our sample.

**Table 1:** Demographic characteristics of participants in subsection of overall sample (*N* = 54).

Participant number	Age	Gender	Ethnicity	Long Covid
Participant 1	20	Female	White	(A) Diagnosed Long Covid
Participant 4	17	Female	White British	(A) Diagnosed Long Covid
Participant 5	20	Male	No response given	(A) Diagnosed Long Covid
Participant 7	17	Female	White British/European	(A) Diagnosed Long Covid
Participant 8	23	Female	White British	(A) Diagnosed Long Covid
Participant 9	25	Female	White British	(A) Diagnosed Long Covid
Participant 10	23	Female	No response given	(C) Describes persistent and ongoing health effects related to Covid‐19
Participant 11	17	Female	White British	(C) Describes persistent and ongoing health effects related to Covid‐19
Participant 13	16	Female	Black/Nigerian	(C) Describes persistent and ongoing health effects related to Covid‐19
Participant 14	20	Female	Chinese	(C) Describes persistent and ongoing health effects related to Covid‐19
Participant 15	17	Male	White British	(A) Diagnosed Long Covid
Participant 16	25	Female	Mixed	(B) Self‐reported, sensed Long Covid
Participant 17	18	Female	Pakistani	(B) Self‐reported, sensed Long Covid
Participant 18	17	Not given	Black British	(B) Self‐reported, sensed Long Covid
Participant 19	16	Female	South Asian, Indian	(C) Describes persistent and ongoing health effects related to Covid‐19
Participant 20	16	Male	Mixed White and Asian	(A) Diagnosed Long Covid
Participant 21	17	Female	South Korean	(C) Describes persistent and ongoing health effects related to Covid‐19
Participant 22	25	Female	White British	(C) Describes persistent and ongoing health effects related to Covid‐19
Participant 23	18	Female	White British	(A) Diagnosed Long Covid
Participant 24	20	Female	Greek Cypriot	(A) Diagnosed Long Covid
Participant 25	18	Female	White British	(B) Self‐reported, sensed Long Covid
Participant 26	18	Female	No response given	(C) Describes persistent and ongoing health effects related to Covid‐19
Participant 27	16	Male	White British	(A) Diagnosed Long Covid
Participant 28	22	Female	White British	(B) Self‐reported, sensed Long Covid
Participant 29	25	Non‐binary	White British	(B) Self‐reported, sensed Long Covid
Participant 30	18	Female	White British	(B) Self‐reported, sensed Long Covid
Participant 32	21	Female	White ‐ Eastern European	(C) Describes persistent and ongoing health effects related to Covid‐19
Participant 34	23	Male	White British	(A) Diagnosed Long Covid
Participant 35	25	Female	White	(A) Diagnosed Long Covid
Participant 36	25	Male	Filipino	(B) Self‐reported, sensed Long Covid
Participant 37	16	Male	White British	(A) Diagnosed Long Covid
Participant 38	25	Female	White	(B) Self‐reported, sensed Long Covid
Participant 39	24	Female	White	(B) Self‐reported, sensed Long Covid
Participant 40	25	Non‐binary Trans Masculine	White British	(A) Diagnosed Long Covid
Participant 42	20	Male	White British	(B) Self‐reported, sensed Long Covid
Participant 43	22	Male	White	(A) Diagnosed Long Covid
Participant 44	17	Female	White British	(B) Self‐reported, sensed Long Covid
Participant 45	20	Female	White British	(B) Self‐reported, sensed Long Covid
Participant 46	23	Female	White British	(A) Diagnosed Long Covid
Participant 47	17	Female	White British	(B) Self‐reported, sensed Long Covid
Participant 48	24	Female	White British	(B) Self‐reported, sensed Long Covid
Participant 49	22	Female	White British	(C) Describes persistent and ongoing health effects related to Covid‐19
Participant 50	23	Female	White British	(A) Diagnosed Long Covid
Participant 51	17	Female	White British/White European	(C) Describes persistent and ongoing health effects related to Covid‐19
Participant 52	18	Female	White British	(A) Diagnosed Long Covid
Participant 53	19	Female	Indian	(C) Describes persistent and ongoing health effects related to Covid‐19
Participant 64	16	Male	White British	(A) Diagnosed Long Covid
Participant 65	22	Male	British	(C) Describes persistent and ongoing health effects related to Covid‐19
Participant 66	19	Male	White British	(A) Diagnosed Long Covid
Participant 67	25	Male	White British	(C) Describes persistent and ongoing health effects related to Covid‐19
Participant 68	22	Male	White British	(A) Diagnosed Long Covid
Participant 69	25	Male	White European	(A) Diagnosed Long Covid
Participant 71	22	Male	White British	(C) Describes persistent and ongoing health effects related to Covid‐19
Participant 72	22	Male	White British	(C) Describes persistent and ongoing health effects related to Covid‐19

### 2.3 Data Generation

The 54 baseline interviews analysed here took place between January and November 2024, lasted on average 63 min (ranging from 42 to 135 min), and were facilitated by Z.C., H.C., P.F. Following consultations with peer researchers to inform our approach, we offered interviews in‐person or online, providing questions in advance and including optional breaks. All participants confirmed informed written consent prior to the interview recording. Participants could reschedule their interview as needed, and had the option of attending with a carer. Two participants were joined by a family member, and one opted to meet in‐person, with the rest (53) meeting online due to preference, geography and illness experience.

Both baseline and follow‐up interviews were semi‐structured and guided by participant responses. Interviews focused on how young people's health, well‐being, and futures have been impacted by the viral impacts of Covid‐19 and the experience of the pandemic. Importantly for this study, this included questions about experiences of illness, access to formal care, and broader support networks. Participants were also asked to reflect on how Long Covid intersects with other conditions or life experiences, including material and social precarity impacts. Follow‐up interviews were conducted with 30 participants to understand how health impacts related to Covid‐19 change over time. Interview questions were tailored to the individual participants but included questions about the long‐term effects of Covid‐19, societal perceptions of Long Covid, and access to care and support. These interviews lasted an average of 63 min (ranging from 40 to 116 min). The same participant decided to do an in‐person interview (with others opting for online), and the same two participants were joined by a family member to support them with the interview.

### 2.4 Analysis

We took grounded theory as a ‘nodal point’ [35] for our data analysis, aiming to stay close to the data and use an iterative process to challenge and develop theoretical ideas around ‘informal care’. We developed a descriptive coding framework, delineating data into key themes of interest. These themes were derived from close analysis sessions with authors Z.C., H.C., T.R. and P.F. Each author analysed interview transcripts, staying close to the text. We developed the coding framework collaboratively through six analysis sessions, focusing on an initial selection of 18 transcripts of analytical interest. H.C. and Z.C. then coded all baseline interview transcripts, using NVivo. Here, we focus primarily on the codes ‘state and formal care’, ‘informal and alternative care’ and ‘navigating (finding and accessing) care’.

**Figure 1:**
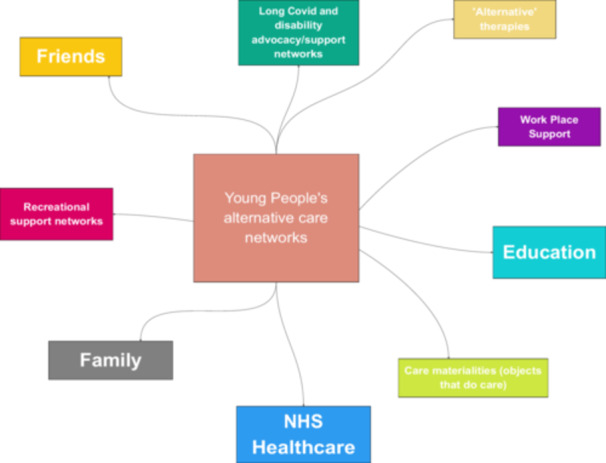
Care networks overview map.

Over three additional analysis sessions, we mapped participants' care networks. In addition to participants' experiences of formal care infrastructures, including healthcare, education and work, we identified six other spheres of alternative care. These include family networks, friendship networks, recreational support networks, ‘alternative’ therapies, Long Covid and disability advocacy and peer support, and the material things, places, and spaces that afford care (see Figure 1).

We also facilitated two specific analysis sessions with our advisory group of peer researchers so that young people could share their views on data extracts and emerging analytical themes. Two peer researchers (L.L. and A.H.) had additional roles in reviewing and feeding into the drafting of this study.

## 3 Findings

In this analysis, we find that young people have had both positive and negative experiences across multiple spheres of alternative care. Rather than describing each sphere of care in the analysis below, we summarise our findings through four cross‐cutting themes that emerged through our group analysis sessions: navigating invalidation of illness experience; the work care networks do; the work needed from young people to make these networks work; and the fragility of these alternative care networks.

### 3.1 Navigating Invalidation

Following previous studies of Long Covid experience [4, 5, 11, 17], participants describe a lack of belief from others in the experiential knowledge they have of their illness when attempting to access care. Young people describe various forms of invalidation, from overt ‘gaslighting’, where symptoms are classed as fabricated, to more insidious or subtle forms of downplaying and dismissal, which can result in being belittled or pathologised, including as mentally unwell:

The doctors at my local hospital at the beginning […] kept trying to push oh, you know, ‘she's a teenager, is she attention seeking?’

[Aged 17, P7]

I remember being sat in this doctor's office and honestly being near tears, because I was like ‘I'm not being believed’ […]. I thought no‐one was ever going to believe me, and maybe this was my fault.

[Aged 17, P4]

Importantly, invalidation is not confined to the medical sphere but materialises across other support infrastructures, including education, work, family and friends:

My dad […] just sort of thought I was lying and wanted to get out of school and I'm like ‘I'm not. I like school, but I can't go in’.

[Aged 18, P23]

At work, so my boss, my employer […] when you're like having to fill in all the forms […] you're putting kind of your medical history or anything like that and so I put Long Covid and he's like, ‘oh I don't believe in Covid anyway so you can leave that off’. I was like ‘okay’…

[Aged 23, P50]

Consequently, young people must explore multiple forms of social connection to find recognition and build care. For some, this was primarily about finding validation, especially when their knowledge had been made unstable by those close to them. P9, for example, describes finding online peer support spaces after experiencing both medical gaslighting and limited belief from her mum:

Facebook groups was my introduction to the fact that there was so many people that also were struggling with this, and that made me feel like really sort of validated.

[Aged 25, P9]

Here, ‘validation’ emerges as a broad theme that extends beyond narrow definitions of medical expertise and diagnosis to include other forms of validating knowledge, including experiential expertise and understanding. It is also for this reason that our advisory group of peer researchers highlighted that ‘validation’ doesn't immediately appear to them as the right word. This is because they do not need an authoritative medical body to ‘prove’ or ‘confirm’ that they are ill, because they *know* this. As P8 says: ‘I knew my body and I knew something wasn't right’. Rather, young people need others to *accept* the knowledge that they have to share about their illness, which in turn can help them access treatments or accommodations. Validation can therefore be afforded in multiple different ways, and according to context, not merely as a confirmation or proof of illness experience, but as a shared connection of understanding, which also contributes to care.

Some young people, therefore, find comfort and recognition through connecting with others with shared bodily knowledge of symptoms and illness trajectories. One way this is accomplished is through online Long Covid spaces. Participants describe engaging with these online networks in different ways, from finding comfort in not being alone in their experience (‘It makes me know I'm not alone’ [P23]), to actively building new connections and sharing experiences. P4, who was ‘taken aback at how little support I'd received in the medical profession’, found support from ‘the more unlikely sources’ of these groups:

The main thing that's really helped has been Long Covid Kids [… which] is providing a bit of a sense of community. […] I looked on there and I found some people who are about my age, going through a similar thing. And so I'm friends with a lot of those.

[Aged 17, P4]

**Figure 2:**
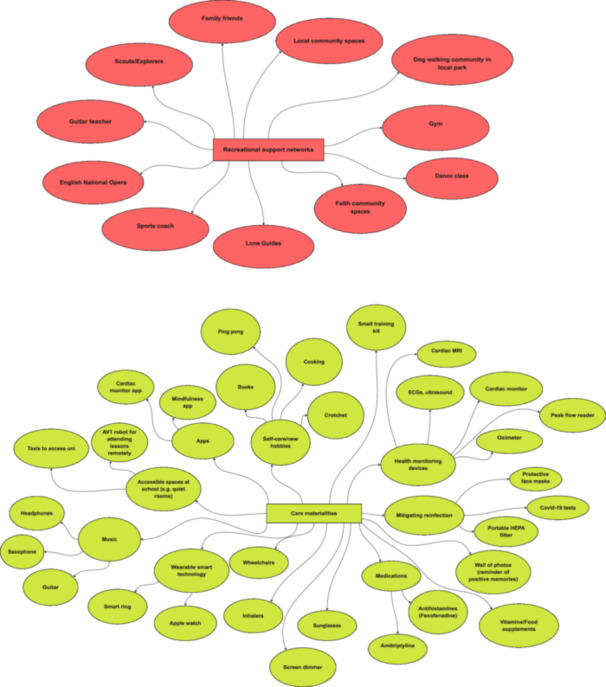
Recreational support networks and care materialities maps.

**Figure 3:**
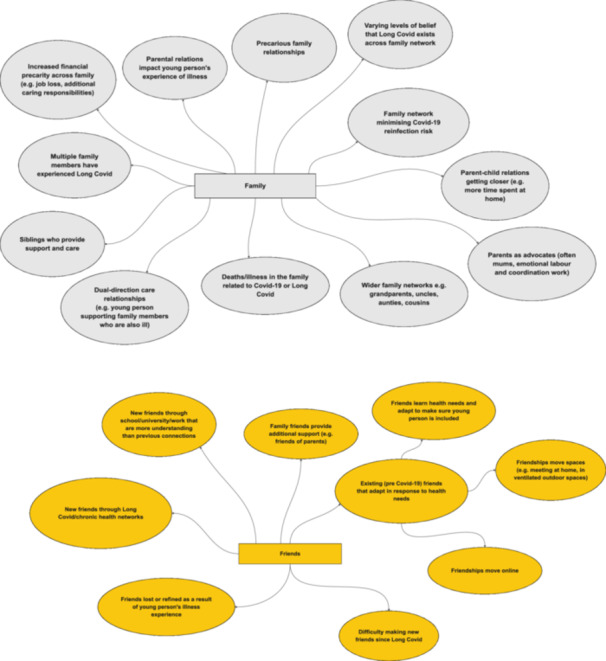
Friends and family network maps.

**Figure 4:**
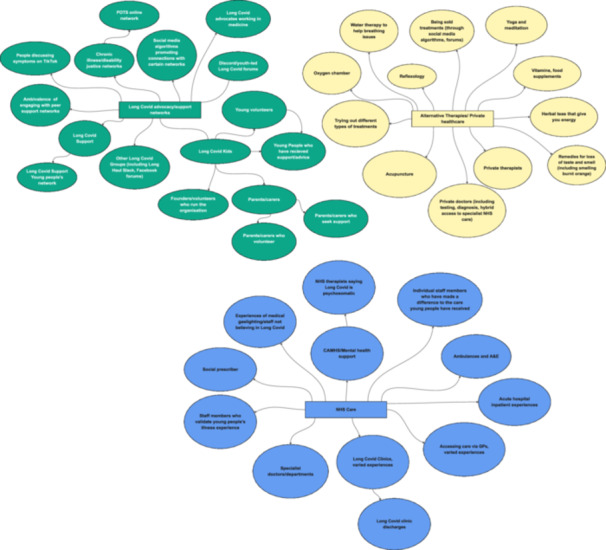
NHS, alternative healthcare and Long Covid peer support maps.

**Figure 5:**
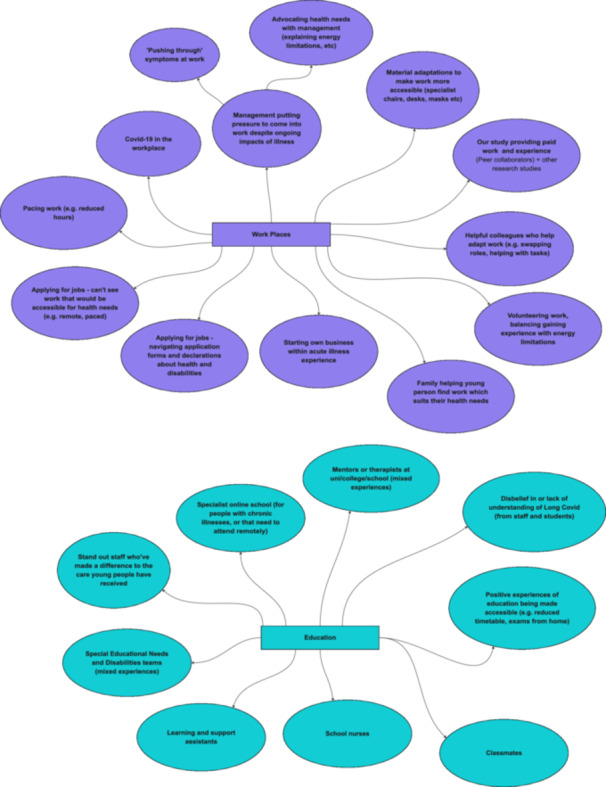
Work and education network maps**.**

Other participants engaged more minimally with online groups, including learning second‐hand from parents who are ‘keeping tabs’ (P37) on these forums. Others decided they didn't want to engage at all (‘I didn't really want to open up about it [Long Covid]’ [P5]). In fact, we find that young people's alternative care networks are much broader than online Long Covid networks alone, encompassing other spheres, including family and friendship networks, local community, and recreational spaces (see Figures 1, 2, 3, 4, 5). These networks may provide forms of recognition or could just be about making spaces for ‘talking through stuff’:

I'm not able to train anymore […] but I've actually got really close with [my sports coach] because he tries really hard to keep me in the loop […] what everybody's doing and like how the clubs going […]. So it's nice to still have that relationship […]. I've got a bit more open about sharing [my poems] as well, […] my [sports] coach as well, he goes, ‘Oh wow, I didn't know like how bad it was’.

[Aged 20, P1]

There are friends of mine from [home], from like that time who are based in such different parts of the country that somehow for the last, God it is over 4 years now, crikey, we've managed to keep up regularly calling doing game stuff online. It doesn't matter where we are in the country or the world we've managed to keep that up […]. It's more talking through stuff than just playing games really.

[Aged 22, P43]

Like the exchange of poems or playing of games, these networks involve more‐than‐human interaction, and also include important materialities that enable care:

What's most consistently helpful is like music, […] mainly guitar [.] and then I've nicked a bass from my guitar teacher and nicked a drum set from my mate […]. I get into sort of like meditative state […] having something really fulfilling that I can actually do, is just really, it's really good for my sort of wellbeing.

[Aged 16, P20]

As P4 explained above, there can be surprises where care can and can't be found across these different networks. Care is not always received where it is expected, and sometimes emerges elsewhere, or through more complex networks:

The lack of support that I had from my, like from health professionals and some of my friends, did impact me, like has impacted my journey massively. I think the most support that I received is actually from my dad, which was surprising […]. Like obviously we're close, but we don't tell each other that. So that was weird to me.

[Aged 23, P46]

I'm a bit surprised, like my dad's friends, if you like, how helpful they've been. […] If they've heard of any clinical trials happening anywhere or anything […] they will always let my dad know so that I can do it. […] There's one of them whose wife works at a hospital somewhere and if there's any new tablets or anything which are available that might help, he lets me know about them so we can look into them.

[Aged 16, P64]

The connections that are made into networks of support are also in flux and can change over time. For P20, the initial ‘very unhelpful’ encouragement from his dad to ‘just keep going’—which de‐legitimised his energy limitations – has since shifted, as ‘he's changed his tune a bit’. Others have found belief and support to wane as their illness persists over time. P27, for instance, explains how the continued presence of his face mask generates ‘really difficult’ comments from school students and staff, as more time elapses from the era of compulsory lockdowns: ‘They sort of say loudly to their friends […] ‘Take off the mask, it's 2024’. As we shall see below, navigating connections across different spheres to enable support takes work—it is a process that young people have to manage over time and given their changing situations.

### 3.2 The Work That Networks Do

The informal networks into which young people connect not only afford validation, in different ways, but also operate as forms of care to facilitate material differences in their lives. One way in which they do this is to help young people locate places where they can receive the treatment they need. For those most ingrained in online Long Covid communities, this often involves finding private doctors or alternative therapies on the recommendations of others:

Mum was in all the like forums and stuff […] but I'm like a little bit ashamed to admit we had to go private […] it's in Liverpool […] [Interviewer: What do they do that's different than the Long Covid Clinic?] So, first off, for risk of being blunt, treat me! Um, you know, action, you know, do something.

[Aged 16, P20]

I've experienced oxygen therapy […] I actually did see an improvement […] that was like when I first could get out the house I was like ‘now I really want to try all the things I've seen on Facebook’, basically.

[Aged 25, P9]

For others, finding diagnosis through online networks, then opens up other networks, such as receiving accommodations at school. Whilst networks may feel separate, in different spheres of life or state infrastructure, they are at once also connected:

My mother decided to like take it upon herself to see if there's anything she could do to help me, so she went on Facebook and found a place called Long Covid Kids […] that's where she found the consultant that actually gave me a kind of diagnosis of Long Covid. […] Now I've got this diagnosis, what they did for my GCSEs was they did put on […] extra time and break during the exams.

[Aged 18, P23]

Whether young people are engaged in online forums or have only heard ‘drips and drabs of bits about Long Covid’ (P68), wider care networks can do important work in making material accommodations and adaptations in people's everyday lives, beyond medical treatments. This involved work from others in their support networks, which include adapting what activities people do in the workplace, enabling young people to see friends, or altering the physical environment of educational settings. These can occur ‘in the moment’, but more often have to be negotiated or learnt as new normalities:

I would do like half a round of nappies, and then someone would take over from that, because I couldn't stand longer, or didn't have the energy to do it […] that was just in the moment.

[Aged 24, P39, nursery worker]

My new manager sat me down, was like, ‘Look, when it comes to the trolleys, do we have a bit of a issue going on here? Because I'm being told sometimes that quite often you're saying no to doing them’ […] I just explained the situation and said, ‘You know, sometimes I'm not going to be able to’ […] there was understanding and they were accommodating of that.

[Aged 22, P68, supermarket worker]

I remember I just passed out in the middle of the gallery and [my friend] was there the whole time, and the best thing about her is that she doesn't kind of take the mick out of it, but she would always just act like it's normal, […] it was nice for someone to be like, just kind of, it was like, ‘okay, well, it's happened, whatever, okay, let's move on now’.

[Aged 18, P52, student]

### 3.3 Making Networks Work

Our findings accentuate that the connections that do work to accommodate young people are not simply found in pre‐existing networks, but also have to be brought together, including in new ways, as well as ‘made to work’, often by young people themselves. Young people must carefully navigate how they piece together complex, fluctuating and unpredictable networks in search of validating relations of care, building knowledge of where recognition and care can be found, and maintaining boundaries where needed. Dealing with fluctuating symptoms and energy levels, leaving ‘less tolerance for bad things’ (P9), many describe strengthening connections with the ‘good people’ (P15), who understand, believe and adapt to their health needs. As P23 encapsulates:

To some people family's important, but to me I feel like it's those who support you most, that are the most important in your life, be them family, friends, teachers, colleagues […] those are the people that you need to start focusing on more. It's like I know blood's thicker than water, but like friendship's better, and support is better than blood.

[Aged 18, P23]

Some people speak of particular ‘stand out’ individuals who help them make material accommodations in work, education and healthcare settings. For many young people, parents and particularly mums, do a lot of work here, with a few people also telling us their ‘mum does a lot of work with Long Covid [charities]’ (P5). However, this also extends to particular teachers, healthcare practitioners and colleagues:

I explained it to [a university tutor] […] He took it and he dealt with the school on my behalf ever since, and if I didn't have that, because that's the point, if I didn't have that, I wouldn't have my degree.

[Aged 25, P69]

There's this sort of like […] constant like power struggle between like the SENCO in the school and the upper management, where the upper management just don't really, you know, care but […] it's the SENCO trying to get them to care […] They're persistent, I'll tell ya.

[Aged 16, P20]

They [the Scout group] continue to be very supportive. They hold meetings outside where they can so I can go and join in and, you know, I don't have to go indoors with them […] they said straight away, ‘yeah, no problem, we'll do that’.

[Aged 16, P27]

Crucially, it is not just people in young people's care and support networks who are doing the work. As emphasised by P20's trip to Liverpool, which was over 200 miles from home, young people must often travel long distances and even stay overnight in order to make these networks work. P50 had a similar experience travelling to London:

So my mum, she was obviously very, very concerned so she was sort of scouring the internet trying to find any Long Covid or any Covid expert […] she found this guy in London […] I go to London sort of every few months […] but it's still like having to pay for a hotel.

[Aged 23, P50]

This illustrates how networks do not simply have to be navigated, but have to be *made*; with connections drawn between people and things accomplished by the work and movement of young people themselves. For P7 this means having to connect up fragmented family, GP, hospital, and educational networks, in order to orchestrate care:

I got out of hospital, I didn't have any education for a year, pretty much, because I had no referrals for a year because my GP messed it up and lost the referral for 6 months, […] they said they couldn't find it in the system, which was bogus because we found the email online and then emailed the Fatigue Clinic ourself and that's how we got the referral done.

[Aged 17, P7]

Importantly, making networks also means severing invalidating, unhelpful, or harmful connections. Some severe connections with ‘gaslighting’ healthcare professionals by finding alternatives or opting out of harmful care. Others re‐evaluate friendship and family connections:

I've gotten rid of more friendships than I've gained […] I'm super protective over myself […] when I notice that like a friendship is like one‐sided […] I'm quite comfortable to move on.

[Aged 25, P9]

[Son] took himself off all social media, you know, because it was just a constant reminder of what could be happening if he was okay and, you know, seeing all his mates going off travelling and going out partying.

[Mum of P66, aged 19]

The young people in our study form tactics in how to navigate making connections or boundaries in their support networks. For instance, in the face of uncertainties in relation to illness invalidation, one tactic used by young people is deciding whether to disclose their Long Covid and with whom. P69, for example, uses the term Long Covid when accessing formal care or with family, but is more cautious across other spheres of his informal care network:

I won't use the term straightaway, until I know them well, because I don't know what they think about it. And even my very best friend […] as I was telling him I was like trying to watch for his reactions, […] you learn […] to guard yourself.

[Aged 25, P69]

Others are still experimenting with how to talk about Long Covid, particularly whilst trying to access work:

When I'm applying for stuff and it's like a 3 year degree and you graduated a year late, I've not really come up with a succinct way to explain it that doesn't also make them think you've got a bunch of health issues and they can't be bothered with you. […] I'm trying to come up with a way to explain it that kind of also leaves it as an issue in the past that's not gonna impact like a job.

[Aged 23, P50]

Developing these tactics to build care networks in difficult contexts does not come without its limitations. Both the making and maintaining of networks can cause fatigue, and even pain, as young people try out different forms of treatments to build up these networks of knowledge:

They [the doctors] are all in London, so we either do Zoom appointments or I have to go up to London to do the appointments. […] it's very fatiguing.

[Aged 16, P27]

It [the private doctor] was very far and not very good for my fatigue but it was something I had to do.

[Aged 18, P23]

We tried most things. If, like when you did your research, if there was like negative side‐effects from it. We kind of weighed it up and generally didn't do those ones. […] I had to try acupuncture at one point which… [Interviewer: And how did you find that?] Painful […] it wasn't beneficial at all. […] [Interviewer: Who recommended acupuncture to you?] I think one of my mum's friends on Facebook who's had experience with Long Covid.

[Aged 16, P64]

Sometimes, even the work of explaining their experience of Long Covid to others can be a lot. As P15 describes how he lost his friends because he wasn't able to explain:

I was trying to explain it to them and that was kind of before I had the reduced timetable in place so I was very tired at breaks and lunch and trying to save as much energy as I could for the other lessons in the day, so I wasn't really doing a very good job of it.

[Aged 17, P15]

The work of self‐advocacy can become a full‐time job, undertaken without institutional recognition or support. Unfortunately for some, it feels that the energy that goes into building these care networks can be more than what they get out of them:

The taste and smell was more experimental. I think I'm probably helping them more than they're helping me. I sort of have to go there and do like smell tests.

[Aged 23, P50]

I first applied [to PIP], or rang up to apply I think in April, so it was a lot of stuff around the same period. The initial application took it out of me, it absolutely exhausted me, and then it was waiting for a month or so, having the needs assessment, that then kind of wiped me out as well.

[Aged 25, P40]

### 3.4 Fragile Networks

Because the very networks that bring about support for young people are made largely by people who are chronically ill and energy‐limited, the networks are often in themselves fragile. The work of sustaining these networks happens alongside the daily work of managing illness, navigating inaccessible systems, and enduring atmospheres which destabilise experiences of Long Covid. This can particularly be seen in online Long Covid advocacy networks, illustrating how connecting with people with similar illness experiences is both fulfilling and precarious, as illness and capacity to engage fluctuate across networks. P4, for example, describes disrupted efforts to raise awareness of Long Covid:

The leader of the charity has just like took a break because of health and stuff, so everyone's very busy trying to keep it afloat […] it makes me feel good that I'm kind of raising awareness and might be helping people […]. Even that's kind of derailed […] it's five kids with Long Covid doing it so a lot of the time it's like ‘sorry, can't do today, I'm ill’.

[Aged 17, P4]

Participants reflected on how the spaces that provide help can also become spaces of exhaustion, requiring a careful rationing of energy:

It's definitely helped and is definitely hard to manage energy wise […] I have to be quite strict, quite time limited on things. […] that's where my extra energy, my brain battery goes […]. You don't really realise what impact that has on you […] some days it can be really difficult.

[Aged 20, P1]

Networks, therefore, reflect the unstable trajectories of Long Covid itself: fluctuating symptoms, unpredictable recoveries and ongoing relapses. At times, this means participants sometimes move on when they recover or find better care. While understandable, these departures can destabilise those still utilising the group and mean the labour of building and maintaining these networks can fall disproportionately on those who remain sickest:

Since I've started to get a bit better […] I don't look at the Facebook groups anymore because they tend to focus on the negative aspect of their journey […] if I surround myself with a lot of negativity, it adds to the fatigue.

[Aged 25, P9]

I kind of stay out of the chronic health forums, these days. I just… [Sighs] I don't think there's anything wrong with them when you are like first unwell and things, but I think after a while they can be more negative to some people than positive because you get lots of people that are just very kind of saying ‘oh, this is terrible’ […] which is fair […] but I think sometimes it's a bit more negative for your mental health.’

[Aged 25, P29]

Like any community, these networks can also reproduce tensions, exclusion, and burnout. Maintaining spaces of care, therefore, requires shifting relations and creating new networks to prevent collapse. As P8 describes:

Some of the initial groups weren't so helpful […] it was very like doom and gloom. […] I found other people who were more closely related to me in age […] and then a group of us started like a younger persons’ community on Discord […] it's a really good space, I'd say that's probably the most helpful.

[Aged 23, P8]

Others illustrate how these online Long Covid networks can also highlight material precarities, reflecting their situatedness in particular socioeconomic conditions [28]. P66's mum, for example, reflects on the alternative options for treatment shared in these spaces:

We sadly don't have money, loads of money spare […] It does annoy me a bit that you know there is all these private doctors that are saying they can see you, but obviously you've got to pay an absolute fortune.

[Mum of P66, aged 19]

While these networks offer lifelines, they are also delicate ecosystems which embody both possibility and precarity. The precarity of these online networks extends beyond, and is in part a product of, fragilities in people's wider support networks. This may mean loss of friends who have ‘kind of disappeared’ (P4), but it also illustrates how illness in social networks can have knock‐on effects in other bodies, meaning care falls short:

The second year at college, they basically said, ‘we don't think you are medically fit, we suggest you withdraw’ […] I did write quite a long email, wasn't it, and said that they hadn't followed their policies and procedures but unfortunately I had pneumonia at the time, so I didn't actually fight too much, on that one.

[Mum of P66, aged 19]

My mum has also taken like a lot of sick leave […] her work has taken a huge hit because she's had to care for me. And it's just been, I think hugely stressful for them, and hugely upsetting for all of us.

[Aged 17, P4]

P66's mum got to a point where she was unable to fight further. In the landscape of Long Covid care, ‘letting go’ is not about giving up lightly, but often reflects the need to survive with limited capacities and exhausted care networks [32]. It can also mean that young people have to patch up their networks, hold them together, and care for those who care for them. Whilst the person doing the research to find P27 medical care has been ‘mainly [his] mum’, he also has to care for her:

If she's feeling particularly fatigued I, I'll make her drinks, I'll make her food. She wears compression stockings but, so I have to, she can't get them off on her own so I have to take those off for her every day […] I help her make dinner in the evenings, I wash up with my sister in the evenings.

[Aged 16, P27]

Young people's family support networks can also be fragile for other reasons; their disbelief in their illness experience, coinciding tensions around lifestyle, gender, or sexuality, or having grown up in care. For some participants, this means finding ways to live with ever‐present invalidation, while others cease attempts to gain understanding from family members, instead concealing the realities of their illness and receiving minimal support:

I had to try and explain to my dad while literally not even being able to sit up, like what he needs to do […] [getting reinfected] is hard, because he doesn't wear a mask when he's out, like me and my mum […] he doesn't truly understand like how hard Long Covid has been for me.

[Aged 17, P7]

My relationship with my mum's been really fractious over the past couple of years […] I'm exhausted and if somebody asks me to do something when I don't have the energy practically to breathe sometimes, I'm going to snap […] and then like I'm kind of doing stuff with… stuff with gender […] so yeah it's been a 10‐year kind of uphill but we're getting to the top of the uphill now, we just need to kind of broach the topic with them at some point.

[Aged 25, P29]

It just sort of I think underscores all the things that care leavers already face like not having the bank of mum and dad […] somewhere to run to […]. I always try and have my own back and carve out the future that I best can […] there is that fear of what if my health like physically or mentally gets in the way of that and I can't work for a little while or I'm not giving it 100%.

[Aged 23, P10]

This illustrates how some people experience more fragile networks than others, because of the different positionalities and spaces they occupy within those networks. This is accentuated by the situatedness of participants in broader systemic networks and infrastructures of money, work, education, and transport links that are themselves fragile or precarious. This can leave participants unable to access healthcare, at risk of reinfection of Covid‐19, or exacerbating symptoms by having to ‘push through’:

It's tight. I mean [my mum] works in the supermarket. It's not good money. I don't, you know, don't qualify for any form of benefit, I don't really have any money, I can't really go and visit people […]. My problem very much is the cost of [transport] by far because it is just extortionate quite frankly […and] a lot of the treatment I was doing for it [Long Covid] was based in [a town in South of England] and it was not something that was particularly transferable down here.

[Aged 22, P43]

Last year [working at a supermarket] we had someone who had Covid, and they said, ‘Look, I'm not going in, I do not want to spread it to my colleagues and other customers’, and due to that he was given an investigation and was ultimately let go […] unless I feel like I really can't go in, I'm just going to have to grin and bear it.

[Aged 22, P68]

Four days a week seems the best balance of trying to see what I can afford living in a city and then, yeah, what I can afford health‐wise as well. […] The workload's quite a lot, I struggle a lot with fatigue [but] all I could afford was 80% part time [laughs].

[Aged 25, P40]

Whilst young people develop, patch up, and hold together support systems for Long Covid, they are often shaped by, and destabilised by, the wider systemic infrastructures in which they are situated. This can mean difficulties in co‐ordinating networks which are fragmented, or contain obstacles to care:

I tried applying for Universal Credit where even though I do not come anywhere close to the cut‐off point for money, not even close to a quarter of their cut‐off point, I was automatically getting rejected for interview for quite a while and I had no idea why at all.

[Aged 22, P43]

Young people also continue to be impacted by the fragility of NHS and state welfare infrastructures, particularly for those unable to access private care. Whilst peer networks can provide advice on how to access formal medical treatment, and even re‐infrastructure and tie together different parts of these systems, they cannot replace all of it:

When I got referred to Cardiology […] they told me it could be an eighteen month wait […] before they actually looked at the piece of paper they were sent, to say my name and why they'd referred me, like not eighteen months till I get an appointment […] which is absolutely shocking […] It upsets me […] it's rubbish like my heart, feeling like it is actually beating out of my chest.

[Aged 18, P30]

My GP did a referral […] and then Long Covid Clinic couldn't take me on […] I was referred to Respiratory Clinic, and Respiratory Clinic said it's a Long Covid issue […] I seemed to just be like, going back and forth, and no‐one really wanted to like take me on.

[Aged 20, P45]

## 4 Discussion

In the face of fragmented care systems, networks of informal support play a vital role. Whilst we could only understand these more intimate networks through spoken accounts rather than ethnographic observation of care as practice, our analysis illustrates the connections that young people draw upon to build and navigate networks of support, enabling their illness experiences to be validated in different ways. Moreover, informal networks can afford care in ways that make material differences in the lives of young people, including in relation to treatments, illness management, and everyday life adaptations and accommodations. Importantly, while *vital*, informal care networks are also *fragile*. Our findings show that networks need to be ‘made to work’. They are not simply identified and found on the basis of pre‐existing connections, but they are also *put together*, assembled across different spheres of connection, as well as *made*, and then *held in place*, by young people. We draw the four themes in our analysis together to contribute to the literature on informal care networks amongst young people living with Long Covid in three ways:

### 4.1 Validation and Care

As other studies have found [5, 11, 16, 36], how Long Covid is recognised and understood by others is a critical element of the illness experience. The invalidation of illness experience that young people have to navigate extends beyond ‘medical gaslighting’, but is a feature of social interactions more broadly—with other young people, with friends and family, with education and work environments and with wider discourses and policies of Covid and pandemic response. Our findings build on those of other studies that have highlighted how people experiencing Long Covid can have their illness experiences dismissed, doubted, or belittled, including by narratives which afford Long Covid weak ‘social currency’ [4]. A key part of the work that young people are doing when piecing together informal networks of support is navigating the invalidation of their illness experience. This highlights that ‘validation’ is more than simple proof or confirmation of illness in relation to medical expertise – something that some young people with Long Covid do not feel they need – but also relates to the creation of connections which enable shared understandings and a sense of collective gathering [17, 27]. Importantly, informal networks not only afford validation in different ways, but also various forms of care. As our findings have illustrated, this includes finding alternative forms of healthcare and treatment for Long Covid, securing accommodations to access education or work, and finding fulfilment in daily life.

### 4.2 More Than Online

Previous research has emphasised the importance of online peer support in providing a space for connection, mutual understanding, and Long Covid advocacy [6, 11, 27]. Our data contributes to the literature by demonstrating how a much broader range of connections make up the alternative care networks that young people draw upon, build, and strengthen in the pursuit of understanding and care. There are surprises in where young people do and do not find care across broad networks, which are entangled and complex. Online Long Covid peer support infrastructures do have an important, though nuanced, role in some young people's networks, amongst other spheres of support, but others have minimal engagement or find connections separate from these spaces. This points to a broader cohort of young people finding alternative routes to access care, beyond those represented in research focused on online communities. Furthermore, this illustrates how differentiated access to social support networks can exacerbate inequalities, as some young people navigate fluctuating symptoms with limited connections to draw from, left to get by with minimal support. While this study did include young people with multiple experiences of marginalisation, further work is needed to include people with experience of racialisation [28].

### 4.3 Work

As illustrated in other chronic health research, creating and sustaining informal networks of care requires ongoing work [32, 33, 34]. Our data finds parallels in relation to Long Covid literature, emphasising the work that young people do to access the validation and care afforded by informal care networks. This work involves bridging across different networks and learning tactics to approach inaccessible systems, with the onus being on young people to make their networks work. Importantly, we illustrate that these connections do not simply exist, but must be worked by young people, who piece together, coordinate, and nurture connections, often holding fragile networks of care together by adapting and rebuilding. Whilst the care networks that young people access and create can be helpful, and bring real material change and accommodations to their lives, these nets of care are forms of work. The everyday demands of being ill can be made long by the extra labour of explaining symptoms, advocating for basic accommodations, coordinating care across fragmented services, and participating in peer networks that can be helpful but also demanding. At times, this work is done by others around young people—their parents or standout individuals they find along the way—but sometimes the amount of work young people have to put into seeking care can take more energy than what they gain from the care they receive.

## 5 Conclusion

In the co‐production of this analysis, peer researchers attest to the work that young people do to hold together their fragile care networks in the face of ‘broken’ state support, which can feel like ‘going round and round a roundabout, but every exit is covered by a roadblock’. The work of informal care is vital, but it should not be celebrated without critical reflection, for it runs the risk of sustaining a precarious situation in which young people are made responsible for picking up the slack and for plugging the gaps of inadequate care. Moreover, alternative care networks do not simply substitute or ‘fill the gaps’ of formal care, but afford different forms of support. Informal care networks can help young people find ways to manage illness and to push for better care in the face of constraints, but this alone is not sustainable or equitable. There is a need to build upon and strengthen the vital work of informal care, and especially the work that young people themselves are doing. But also, there is a need to work with young people to co‐produce policy and services that properly integrate formal and informal care infrastructures that are not only validating but that provide care in direct response to young people's illness experiences.

## Author Contributions

**Zaira Clarke:** writing – original draft, writing – review and editing, formal analysis, conceptualisation. **Hannah Cowan:** writing – original draft, writing – review and editing, formal analysis and conceptualisation. **Tim Rhodes:** writing – review and editing, formal analysis and conceptualisation, funding acquisition. **Praveena Fernes:** writing – original draft, writing – review and editing, formal analysis and conceptualisation. **A. Haines:** review and editing. **Lucinda Leal:** review and editing.

## Ethics Statement

This research received ethics approval from the London School of Hygiene and Tropical Medicine Observational Research Ethics Committee (Reference: 28635).

## Consent

All participants interviewed as part of this study have given their consent, in keeping with the project ethics approvals.

## Conflicts of Interest

The authors declare no conflicts of interest.

## Data Availability

Data not made available via any data repository raises concerns regarding deductive disclosure.
